# Rheological Behavior of Oil Well Cement Slurries with Addition of Core/Shell TiO_2_@SiO_2_ Nanoparticles—Effect of Superplasticizer and Temperature

**DOI:** 10.3390/ma18020239

**Published:** 2025-01-08

**Authors:** Giovanni dos Santos Batista, Francisca Puertas, Antonio Shigueaki Takimi, Eleani Maria da Costa, Marta Palacios

**Affiliations:** 1 School of Technology, Pontifical Catholic University of Rio Grande do Sul (PUCRS), Avenida Ipiranga, 6681, Porto Alegre 90619-900, Brazil; giovanni.batista@edu.pucrs.br (G.d.S.B.); eleani@pucrs.br (E.M.d.C.); 2 Eduardo Torroja Institute for Construction Sciences (IETcc-CSIC), C/Serrano Galvache, 4, 28033 Madrid, Spain; 3 School of Engineering, Federal University of Rio Grande do Sul (UFRGS), Avenida Bento Gonçalves, 9500, Porto Alegre 91540-000, Brazil; antonio.takimi@gmail.com

**Keywords:** oil well cementing, rheology, polycarboxylate ether, temperature, optimization

## Abstract

This study investigates the rheological behavior of oil well cement pastes (OWCPs) modified with core/shell TiO_2_@SiO_2_ (nTS) nanoparticles and polycarboxylate-ether (PCE) superplasticizers at different temperatures (25, 45, and 60 °C). Results show that nTS particles increased static and dynamic yield stresses and the apparent viscosity of the cement slurries due to an increased solid volume fraction and reduced free water availability. The increase in the slurry dispersion by adding PCE superplasticizers enhanced the effect of the nanoparticles on the rheological parameters. Oscillation rheometry demonstrated that nTS nanoparticles enhanced the structural buildup, while PCE retarded hydration. Furthermore, slurries hydrated at 60 °C experienced higher initial values of the elastic modulus and a faster exponential increase in this rheological parameter due to the acceleration of the cement hydration.

## 1. Introduction

Rheological properties of cementitious materials play a key role in the petroleum industry [1]. Before pumping into the wellbore, the cement slurry is mixed on the rig in a cementing unit [2]. During the oil well cementing operations, the cement slurry fills the annulus space between the rock formation and the steel casing. The main objectives of the cement sheath are to provide zonal isolation from fluid flow and to protect the steel casing, preventing it from corrosion [3]. Moreover, if the well presents no zonal isolation, it will never reach its best potential as an oil or gas producer [2]. Many problems can be found during cementing operations, such as improper casing centralization, poor bonding between cement formation and cement casing, shrinkage, the inappropriate placement of cement, and fluid loss [1]. Among them, channeling is the most critical one [4,5].

The study of rheological properties of cementitious materials involves the identification of the inherent properties of fluids, including plastic viscosity, yield stress, frictional behavior, and gel strength. Understanding these factors is crucial in determining the flow behavior of cement slurry and establishing the relationship between the flow rate (shear rate) and the pressure gradient (shear stress) that drives fluid movement [6]. Some studies have shown that, in the early stages of insufficient cement hydration, volume shrinkage and external loads contribute to the deformation of the cement stone, leading to oil and gas well leakage [7,8]. As a result, it is essential to develop oil well cement with suitable rheological properties, a dense hydration product structure, and high early strength. The rheology of the cement slurry is affected by many factors, such as the chemical composition of cement, solid volume fraction, the specific surface area of cement, the shape of cement grains, the type and dosage of chemical admixtures, application methods, the mixing time and process, the nature of wellbore, etc. [9]. According to Nelson and Guillot [6], the effect of pressure on the rheological properties of oil well cement can be neglected as its effect is too small when compared to the temperature. Temperature has a major impact on cement slurry rheology, but it also depends on the incorporated chemical admixtures.

In recent years, nanoparticles have shown great potential to enhance the properties of cementitious materials, such as the heat of hydration, mechanical strength, microstructure, shrinkage, and chemical resistance [10,11]. In terms of hydration kinetics, they can accelerate cement hydration by creating additional nucleation sites for the precipitation and increase of calcium silicate hydrate (C-S-H) gel content. This acceleration of hydration reduces thickening time, facilitating early strength development [12,13].

However, nanoparticles can negatively influence the viscosity of cement-based materials. Traditional cement without nanoparticles has lower viscosity, leading to a more brittle cement bond. In contrast, nano-enhanced slurries exhibit higher viscosity due to a solid/volume ratio increase and, in many cases, a high water adsorption of water used in the paste preparation, which enhances their flexural performance [14]. The addition of nanoparticles, due to their large surface area, typically reduces the workability of cement slurry. Depending on the type and concentration of the nanomaterial, the slurry’s rheology can change significantly, potentially making it unpumpable [15].

The addition of nanosilica to oil well cement slurry alters its rheological properties, such as yield stress and plastic viscosity. Its large surface area increases water and chemical admixture requirements for workability. At constant water content, higher nanosilica concentrations lead to higher solid volume fractions and particle packing, reducing free water and increasing torque and friction between particles [16]. This reduces cohesion, lowering flow spread and increasing plastic viscosity. Nanosilica particles densify the microstructure by filling voids, though agglomerates may retain free water, reducing workability. Whether nanosilica affects rheology depends on whether agglomerates act as fillers [17]. Nanoparticles in water tend to agglomerate due to van der Waals interactions, making it challenging to achieve a uniform dispersion when preparing cement nanocomposites [18].

Baragwiha et al. [19] investigated the addition of nano-SiO_2_ (15–25 nm) and nano-TiO_2_ (20–30 nm) to oil well cement class G in 1, 3, 5, and 7% by weight of cement (BWOC). Polycarboxylate-ether (PCE) superplasticizer and defoamer dosages were kept constant at 1.6 and 0.2% BOWC, respectively. To avoid instant thickening, the w/b ratio varied from 0.72 to 0.78, depending on the density of the slurries, which was maintained at 1.65 g·cm^−3^. The authors observed that nano-SiO_2_ significantly increased the apparent viscosity and yield stress of the slurries. This behavior was attributed to the increased structure building ability of the cement slurries. The effect of nano-TiO_2_ was smaller. This was because, while the effect of nano-TiO_2_ is purely physical (i.e., more nucleation sites due to a high surface area), nano-SiO_2_ also promoted chemical reactions with calcium hydroxide to form additional C-S-H due to its pozzolanic nature.

To improve properties both in the fresh and hardened state, core/shell TiO_2_@SiO_2_ (nTS) nanoparticles are a good alternative [20,21]. This material consists of a mixture of a non-reactive material (which is the core) covered by a pozzolanic material (being the shell). In previous studies [22,23], we investigated the dispersion of TiO_2_ covered with SiO_2_ (nTS) nanoparticles of 20–40 nm in water. After adding 1% BWOC of nTS in water, the mean hydrodynamic size of particles was 32.66 nm, indicating that no agglomeration occurred. Additionally, we studied the impact of 0.5% and 1% BWOC of these particles on oil well cement class G hydrated pastes, in the presence and absence of superplasticizers. A decrease in porosity and portlandite content, increased hydration and C-S-H main chain length, and an improvement in mechanical and chemical properties before and after CO_2_ exposure were observed. No studies on the rheological behavior of these pastes with PCE have been carried out to date. It is therefore necessary to study the influence of these nTS nanoparticles on the rheology of oil well cement slurries and the effect of temperature in these mixtures. For this reason, the main objective of the present study is to investigate the influence of these nTS particles on the rheological behavior of oil well cement class G slurries in the absence and presence of PCE superplasticizers and at different temperatures.

## 2. Materials and Methods

### 2.1. Materials

The cement class G was provided by Lafarge Holcim Brasil S.A., (Rio de Janeiro, Brazil) and its chemical composition was determined by XRF, as presented in Table 1.

The commercial core/shell TiO_2_@SiO_2_ nanoparticles were purchased from SkySpring Nanomaterials. The core consists of rutile phase titanium oxide (TiO_2_), which is 99.5% pure. The core is coated with a layer (shell) of silicon oxide (SiO_2_). The mean nanoparticle size is 32 nm, measured by Dynamic Light Scattering (DLS) [22]. The TiO_2_/SiO_2_ ratio is approximately 0.03, and the nanoparticles are highly hydrophilic. The transmission electron microscopy (TEM) image and the X-ray diffraction (XRD) spectrum of nTS are shown in Figures S1 and S2 of the Supplementary Materials, respectively. The amounts of nTS addition were 1, 2, 3, and 4% BWOC. To maintain good workability, a PCE superplasticizer was used (CQ Plast MR 825 superplasticizer admixture provided by Camargo Química S.A., Pomerode, Brazil). This is a polycarboxylate-ether superplasticizer of the third generation and follows the standard C494 [24] as a type F. The Fourier transform infrared spectroscopy (FTIR) of PCE is shown in Figure S3 of the Supplementary Materials. The physical properties of the powders are presented in Table 2.

### 2.2. Mixing Procedures

The mixing proportions of oil well cement pastes (OWCPs) are presented in Table 3. Prior to mixing, the nTS content was added to deionized water. The amount of cement was 100 g, and the w/b ratio was 0.44 for the mixtures without PCE. For the mixtures with the PCE addition, the dosage of PCE used was 0.15 and 0.30% BWOC, and the w/b ratio was reduced to 0.35 to avoid bleeding. Slurries were mixed at 2000 rpm for 60 s. The rheology tests were carried out in a Kinexus Ultra+ rheometer from Netzsch (Selb, Germany).

### 2.3. Rheological Test Conditions

#### 2.3.1. Static Yield Stress

The static yield stress was determined from the stress growth test at 25 °C. To ensure repeatability, a preshear of 200 s^−1^ was applied for 35 s. The stabilization time was set to 10 min for structure formation. A constant shear rate of 0.1 s^−1^ was applied, and, with the structure breakdown, the shear stress increased. The static yield stress value was obtained through the initial peak of the shear stress (Figure S5 in the Supplementary Materials).

#### 2.3.2. Flow Curves and Apparent Viscosity

Flow curves were measured on pastes at 25, 45, and 60 °C using concentric cylinders geometry with roughed surfaces. A preshear was initially conducted at 200 s^−1^ for 35 s. A stabilization time of 5 min to reach the target temperature was applied afterward. The upper and lower ramps were from 0 to 100 s^−1^ during 2 min and 15 s each. The dynamic yield stress was determined using the Herschel–Bulkley model (Equation (1)), which presented the best fit.

(1) $$\tau =\tau_{0}+K\;{\dot{\gamma }}^{n}$$

where *τ* is the shear stress, *τ*_0_ is the dynamic yield stress, *K* is the consistency factor, $\dot{\gamma }$ is the shear rate, and *n* is the flow index.

The evolution of the apparent viscosity with shear rate was obtained from the down curve.

#### 2.3.3. Oscillation Tests

Oscillation measurements were conducted to study the structural build-up of the slurries at 25 °C and 60 °C and dosages of PCE and nTS. For this, the plate–plate geometry with rough surfaces was used, and the gap between plates was set at 1 mm. The linear viscoelastic region (LVER) was initially determined as this is the domain where the microstructure of the cement paste is preserved. The LVER was determined by increasing the strain at a frequency of 1 Hz [25,26]. For this, small amplitude oscillatory shear (SAOS) tests were conducted at a strain of 5 × 10^−5^ up to 2.5 h. To avoid water evaporation during the measurement, a solvent trap system was used.

## 3. Results and Discussion

In oil well cementing, understanding rheological properties like static and dynamic yield stresses, apparent viscosity, and oscillation tests is crucial for ensuring the performance and stability of cement slurries. Static and dynamic yield stresses help assess the slurry’s ability to resist flow under static and dynamic conditions, impacting placement and preventing fluid migration. Apparent viscosity measures flow resistance, influencing pumpability and placement efficiency. Oscillation tests help evaluate the viscoelastic properties, providing insight into the transition from slurry to solid, crucial for zonal isolation and well integrity. Understanding these properties ensures efficient cementing, preventing well failures, and optimizing production.

In the present study, the nTS content, temperature, and the presence and absence of PCE have been considered as variables in the rheological behavior of oil well cement slurries. The results obtained considering the aforementioned variables are described and discussed below, specifically the following: (i) the impact of nTS and PCE on the static and dynamic yield stress of OWCPs at room temperature; (ii) the impact of temperature and nTS on the dynamic yield stress and apparent viscosity of oil well cement pastes; and (iii) the study of the reactivity of oil well cement pastes containing nTS by oscillation rheometry.

### 3.1. Impact of nTS and PCE on the Static and Dynamic Yield Stress of Oil Well Cement Pastes at Room Temperature

**Figure 1:**
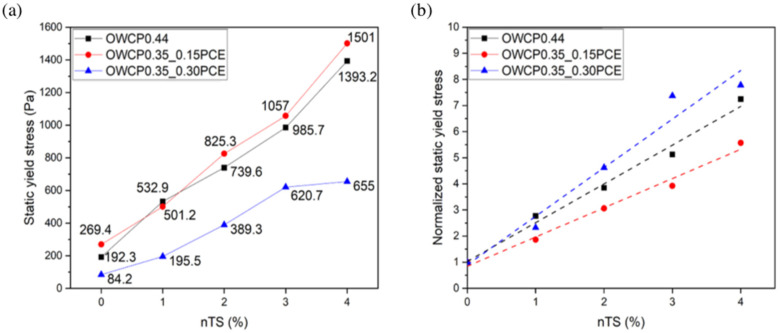
(**a**) Static yield stress values and (**b**) normalized static yield stress at 25 °C.

The static yield stress of the studied OWCPs at 25 °C is shown in Figure 1. This parameter was measured by shear stress-controlled protocols. Static yield stress is the needed stress to initiate flow, and it is associated with a non-disturbed microstructure [27,28].

The addition of 0.15% PCE admixtures enabled the decrease in the water/binder ratio from 0.44 to 0.35 with an increase of around 25% of the static yield stress, regardless of the amount of nTS added to the pastes. This effect of PCE is due to the steric hindrance induced by the adsorbed polymer onto the cement particles [29,30]. The increase in the PCE dosage up to 0.30% led to a decrease of 75% of the static yield stress in the absence of nTS, and this positive effect is preserved when the nanoparticles are added. To compare the impact of nTS in the three cementitious systems, with different water content and polymer dosages, static yield stress values were normalized with respect to the yield stress of the corresponding paste without nTS. From Figure 1b, it can be inferred that the addition of nTS leads to a higher increase in the static yield stress in OWCPs with a l/s = 0.35 and containing the highest amount of PCE, showing that the nanoparticles have a greater effect as the dispersion of particles in the paste increases. This agrees with previous studies that proved that good dispersants are needed to stabilize nanoparticles, such as nano clays or graphene oxide, and preserve their properties as hydration accelerators or viscosity-modifying admixtures [31]. At this early reaction time (around 15 min), the amount of hydrates can be considered neglectable, and the increase in the static yield stress induced by nTS can be mainly explained by the increase in the solid volume fraction of the pastes, as shown in Table 3 [32].

### 3.2. Impact of the Temperature and nTS on the Dynamic Yield Stress and Apparent Viscosity of the Oil Well Cement Pastes

**Figure 2:**
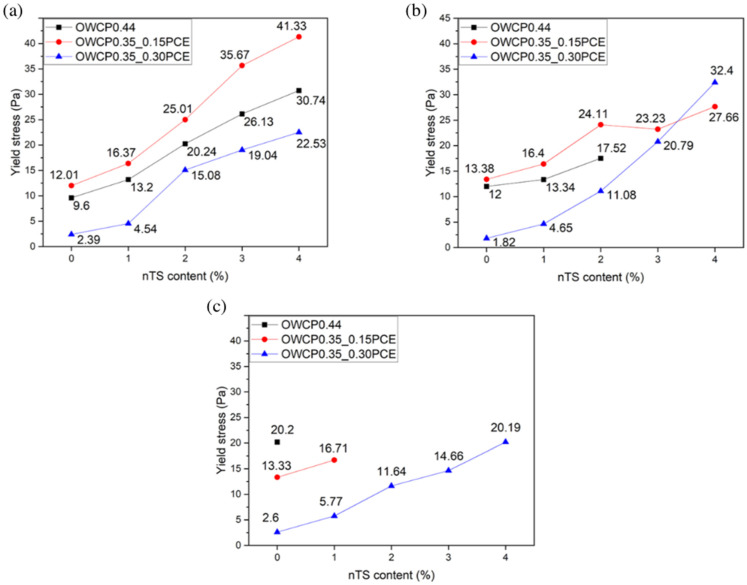
Dynamic yield stress values at different temperatures (**a**) 25 °C, (**b**) 45 °C, and (**c**) 60 °C.

The effect of temperature on dynamic yield stress in the OWCP slurries (with nTS and PCE) is shown in Figure 2. The dynamic yield stress, determined from the flow curve (see Figures S6, S7 and S8a,c,e in the Supplementary Materials), is defined as the minimum stress to maintain or finish the flow of a material that has suffered the breakdown of the microstructure [33,34]. Furthermore, the apparent viscosity of the slurries at different temperatures (with different nTS content and PCE) is presented in Table 4. All the slurries show a shear-thinning behavior with a decrease in the apparent viscosity as the shear rate increases (see Figures S6, S7 and S8b,d,f in the Supplementary Materials).

The addition of nTS increased both rheological parameters, dynamic yield stress, and apparent viscosity compared to the control samples due to the increase in the solid volume fraction (see Table 4) and the water adsorption by nTS that decreases the amount of free water needed for flowability [35,36]. For a given nTS content and temperature, mixtures with PCE tend to present lower values of both rheological parameters as the adsorption of PCE onto the cement surface decreases the attractive interparticle forces that prevent particle flocculation and releases the free water trapped within the flocs [37].

The increase in the temperature from 25 °C to 45 °C does not significantly impact the values of dynamic yield stress and apparent viscosity; however, the low flowability of most of the OWCPs and OWCP0.35_0.15PCE pastes at 60 °C did not allow us to measure their rheological parameters. This is mainly related to the fast reactivity of the pastes at this temperature. In contrast, all OWCP0.35_0.30PCE pastes could be tested at 60 °C, as in this case, the higher dosage of PCE led to a higher amount of polymer adsorbed on the surface reactive areas of the silicate phases and the consequent retardation of cement hydration [38,39].

**Figure 3:**
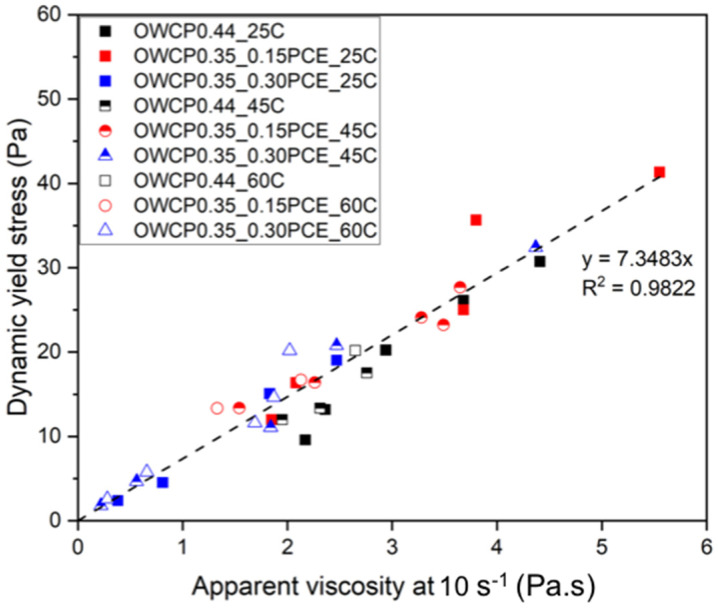
Dynamic yield stress and apparent viscosity relationship.

The dynamic yield stress has been plotted versus the apparent viscosity at 10 s^−1^ for most of the studied pastes, containing variable amounts of nTS, PCE, and hydrated at different temperatures. The master curve obtained (Figure 3) exhibits a linear relationship with a high regression coefficient (R^2^ = 0.9822), showing a strong correlation between dynamic yield stress and apparent viscosity. This further confirms that the observed changes in rheological behavior can be attributed primarily to the physical effect of added solids rather than chemical interactions associated with hydration. In contrast, the rapid flow loss suffered by OWCPs containing nTS and OWCP0.35_0.15PCE with dosages of nTS above 1% and hydrated at 60 °C did not allow us to measure their rheological properties. For these pastes, flow loss was related to the enhancement of cement hydration induced by temperature and the presence of nanoparticles, as also shown below in the oscillation measurements.

These results show that the addition of PCE is essential to maintain low dynamic yield stress values in slurries containing nTS at all tested temperatures. In particular, the synergistic effect of PCE and nTS improved the flowability of the mixtures with low nTS or high nTS with high PCE contents even at elevated temperatures, being beneficial for oil well applications.

### 3.3. Study of the Reactivity of the Oil Well Cement Pastes Containing nTS by Oscillation Rheometry

**Figure 4:**
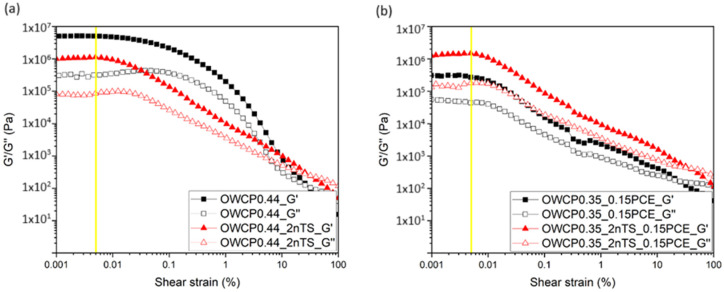
Strain oscillation test conducted on slurries at 25 °C (**a**) without PCE and (**b**) with PCE.

**Figure 5:**
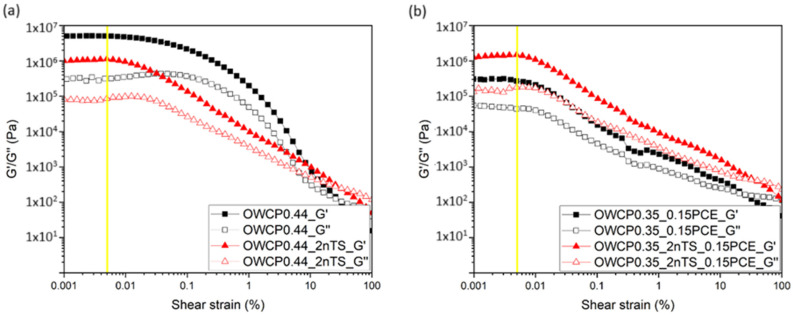
Strain oscillation test conducted on slurries at 60 °C (**a**) without PCE and (**b**) with PCE.

To further investigate the reaction and structuration of the cement pastes, small amplitude oscillatory shear (SAOS) measurements were conducted. The LVER graphs at 25 and 60 °C are presented in Figure 4 and Figure 5, respectively. The range of strain and stress within the viscoelastic properties of the material remains constant, regardless of the applied strain. Until the yellow line, the material responds predictably to strain and stress and can return to its original shape. The critical strain was found in the range between 1 × 10^−5^ and 5 × 10^−4^ Pa. This region was identified immediately before the G′/G″ values started to decrease and was 5 × 10^−3^%.

**Figure 6:**
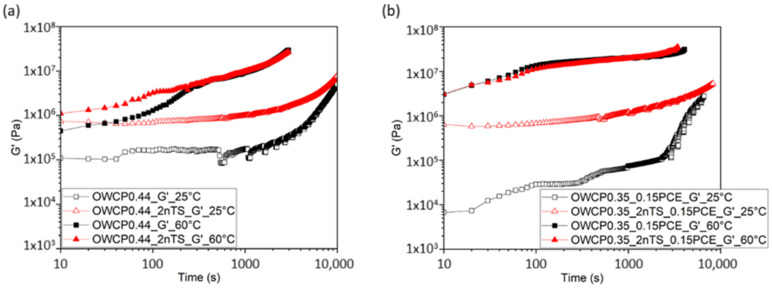
Complex elastic modulus of (**a**) OWCP0.44 and (**b**) OWCP0.35_0.15PCE at 25 and 60 °C.

The SAOS was carried out for OWCPs without and with 0.15% of PCE and dosages of nTS up to 2% [40]. Figure 6 shows the evolution of the elastic modulus G′ over the reaction time at a constant shear strain at 0.005% (value in the linear viscoelastic regime). At initial times, the addition of PCE decreases the elastic modulus of the slurries with respect to the non-admixed one, which confirms the dispersing properties of the PCE superplasticizer. In particular, at 25 °C, OWCP and OWCP0.35_0.15PCE showed a G′ of 1.09 × 10^5^ and 6.7 × 10^3^ Pa, respectively. In contrast, at 60 °C, no significant differences in the G′ values have been observed for both pastes.

The addition of 2% nTS to OWCP0.44 increases the initial elastic modulus compared to non-admixed slurries, inferring that the rigidity of the pastes rises with the increase in the solid volume ratio and the consequent decrease in the interparticle distance [41]. At 25 °C, the elastic modulus remains almost constant over the first 15 min of hydration. Afterward, a steep rise in the elastic modulus is observed probably due to the gradual precipitation of reaction products onto the pseudo-contact points between cement particles [25]. The addition of nanoparticles does not modify the time at which G′ increases, but it reduces the slope of the curve after 15 min of hydration. The rise in the temperature to 60 °C in OWCP0.44 slurries with and without nanoparticles leads to an increase in G′ from the very early times due to the acceleration of hydration kinetics with respect to slurries hydrated at 25 °C.

In the presence of PCE, two main stages were observed in the evolution of G′ of pastes hydrated at 25 °C. Over the first stage up to 2000 s, the progressive increase in G′ is associated with the initial flocculation of the cement particles due to colloidal interactions and the initial precipitation of first hydration reaction products [25,26]. Afterward, an exponential increase in G′ is observed and related to the higher amounts of these reaction products over this stage. The addition of the polymer retards the time of appearance of this exponential increase with respect to OWCP0.44 pastes [39] as the polymer adsorption onto the surface reactive sites blocks silicate dissolution. In slurries containing PCE, the rise in the temperature leads to a higher initial G′ value and a faster increase in this parameter; however, the presence of nanoparticles does not modify the evolution of G′ over time, as shown in Figure 6b.

## 4. Conclusions

This study evaluated the impact of core/shell TiO_2_@SiO_2_ (nTS) nanoparticles and polycarboxylate-ether (PCE) superplasticizer on the rheological behavior of oil well cement pastes (OWCPs) at different temperatures. The findings reveal that nTS effectively changes the rheological properties of cement slurries by increasing static and dynamic yield stresses and the apparent viscosity. This behavior is attributed to an increase in solid volume fraction and a reduction in free water availability. The increase in these rheological parameters induced by the nTS is higher as the PCE dosages increase, which confirms the greater effect of nanoparticles in well-dispersed systems.

The increase in the temperature up to 45 °C induced no significant changes in the rheological parameters. However, slurries hydrated at 60 °C experienced higher initial values of the elastic modulus (measured by oscillation rheometry) and a faster exponential increase in this rheological parameter.

The synergistic interaction between nTS and PCE emerged as a key finding. While nTS enhanced the rheological performance by improving microstructure and particle packing, PCE ensured dispersion and prevented agglomeration, enhancing the overall flow properties of the slurries. This combination demonstrates significant potential for improving oil well cement performance in high-temperature environments.

## Figures and Tables

**Table 1:** Cement class G chemical composition.

Oxide	SiO_2_	CaO	Al_2_O_3_	Fe_2_O_3_	MgO	SO_3_	K_2_O	Equivalent Na_2_O	LOI ^1^	IR ^2^
wt.%	21.1	64.2	3.8	4.6	1.3	2.6	0.4	0.5	0.8	0.53

^1^ Loss of ignition. ^2^ Insoluble residue.

**Table 2:** Characteristics of materials.

Material	Size	Specific Mass (g·cm^−3^)	Specific Surface Area (m^2^·g^−1^)
Cement class G	7.30 µm ^1^	3.16	0.82 ^1^
nTS	32 nm ^2^	4.23 ^3^	~40

^1^ Measured by BET (Figure S4). ^2^ Measured by DLS in our previous work [22]. ^3^ Provided by the supplier.

**Table 3:** Mix compositions.

Mixture	Cement (g)	Water (g)	nTS (g)	PCE (g)	Solid/Volume Fraction (%)
OWCP0.44_0nTS	100	44	0	0	41.9
OWCP0.44_1nTS	100	44	1	0	42.1
OWCP0.44_2nTS	100	44	2	0	42.3
OWCP0.44_3nTS	100	44	3	0	42.5
OWCP0.44_4nTS	100	44	4	0	42.7
OWCP0.35_0nTS_0.15PCE	100	35	0	0.15	47.6
OWCP0.35_1nTS_0.15PCE	100	35	1	0.15	47.8
OWCP0.35_2nTS_0.15PCE	100	35	2	0.15	48.0
OWCP0.35_3nTS_0.15PCE	100	35	3	0.15	48.2
OWCP0.35_4nTS_0.15PCE	100	35	4	0.15	48.4
OWCP0.35_0nTS_0.30PCE	100	35	0	0.30	47.6
OWCP0.35_1nTS_0.30PCE	100	35	1	0.30	47.8
OWCP0.35_2nTS_0.30PCE	100	35	2	0.30	48.0
OWCP0.35_3nTS_0.30PCE	100	35	3	0.30	48.2
OWCP0.35_4nTS_0.30PCE	100	35	4	0.30	48.4

**Table 4:** Apparent viscosity values at 10 s^−1^ at different temperatures.

Temperature	nTS Content (%)	Apparent Viscosity (Pa·s)		
		OWCP0.44	OWCP0.35_0.15PCE	OWCP0.35_0.30PCE
25 °C	0	2.17	1.85	0.38
	1	2.36	2.08	0.81
	2	2.94	3.68	1.83
	3	3.68	3.80	2.47
	4	4.41	5.55	5.17
45 °C	0	1.95	1.54	0.22
	1	2.31	2.26	0.56
	2	2.76	3.28	1.84
	3	-	3.49	2.47
	4	-	3.65	4.37
60 °C	0	2.65	1.33	0.28
	1	-	2.13	0.66
	2	-	-	1.69
	3	-	-	1.87
	4	-	-	2.02

“-” Measurements could not be taken due to the low flowability of the pastes.

## Data Availability

The original contributions presented in this study are included in the article/Supplementary Materials. Further inquiries can be directed to the corresponding authors.

## Supplementary Materials

The following supporting information can be downloaded at https://www.mdpi.com/article/10.3390/ma18020239/s1, Figure S1: TEM image of nTS; Figure S2: XRD spectrum of nTS; Figure S3: FTIR spectrum of PCE; Figure S4: Isothermal graph of anhydrous cement class G; Figure S5: Shear stress graphs for static yield stress determination at 25 °C for the mixtures (a) without PCE, (b) with 0.15 wt.% of PCE, and (c) with 0.30 wt.% of PCE; Figure S6: Shear stress and shear viscosity graphs for the mixtures (a,b) without PCE, (c,d) with 0.15 wt.% of PCE, and (e,f) with 0.30 wt.% of PCE; Figure S7: Shear stress and shear viscosity graphs at 45 °C for the mixtures (a,b) without PCE, (c,d) with 0.15 wt.% of PCE, and (e,f) with 0.30 wt.% of PCE; Figure S8: Shear stress and shear viscosity graphs at 60 °C for the mixtures (a,b) without PCE, (c,d) with 0.15 wt.% of PCE, and (e,f) with 0.30 wt.% of PCE.
