# Supplementary material for: Rheological Behavior of Oil Well Cement Slurries with Addition of Core/Shell TiO2@SiO2 Nanoparticles—Effect of Superplasticizer and Temperature
Source: Materials (Basel). 2025 Jan 8;18(2):239. doi: 10.3390/ma18020239 (PMC11766691; doi:10.3390/ma18020239)
Supplement: Supplementary file 1 [file materials-18-00239-s001.zip › materials-3371601-supplementary.pdf]

Supplementary Materials

Rheological Behavior of Oil Well Cement Slurries with Addition of Core/Shell $TiO_2@SiO_2$ Nanoparticles—Effect of Superplasticizer and Temperature

Giovanni dos Santos Batista [1], Francisca Puertas [2,*], Antonio Shigueaki Takimi [3], Eleani Maria da Costa [1] and Marta Palacios [2,*]

1. School of Technology, Pontifical Catholic University of Rio Grande do Sul (PUCRS), Avenida Ipiranga, 6681, Porto Alegre 90619-900, Brazil; giovanni.batista@edu.pucrs.br (G.d.S.B.); eleani@pucrs.br (E.M.d.C.)
2. Eduardo Torroja Institute for Construction Sciences (IETcc-CSIC), C/Serrano Galvache, 4, 28033 Madrid, Spain
3. School of Engineering, Federal University of Rio Grande do Sul (UFRGS), Avenida Bento Gonçalves, 9500, Porto Alegre 91540-000, Brazil; antonio.takimi@gmail.com

* Correspondence: puertasf@ietcc.csic.es (F.P.); marta.palacios@ietcc.csic.es (M.P.)

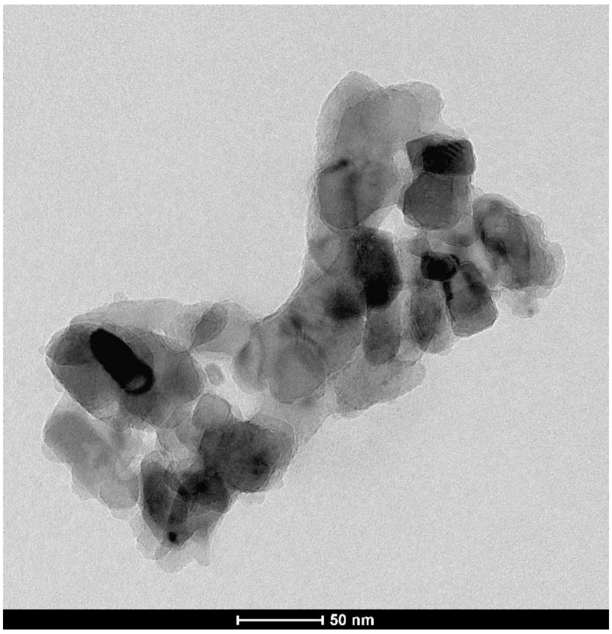

Figure S1. TEM image of nTS nanoparticles.

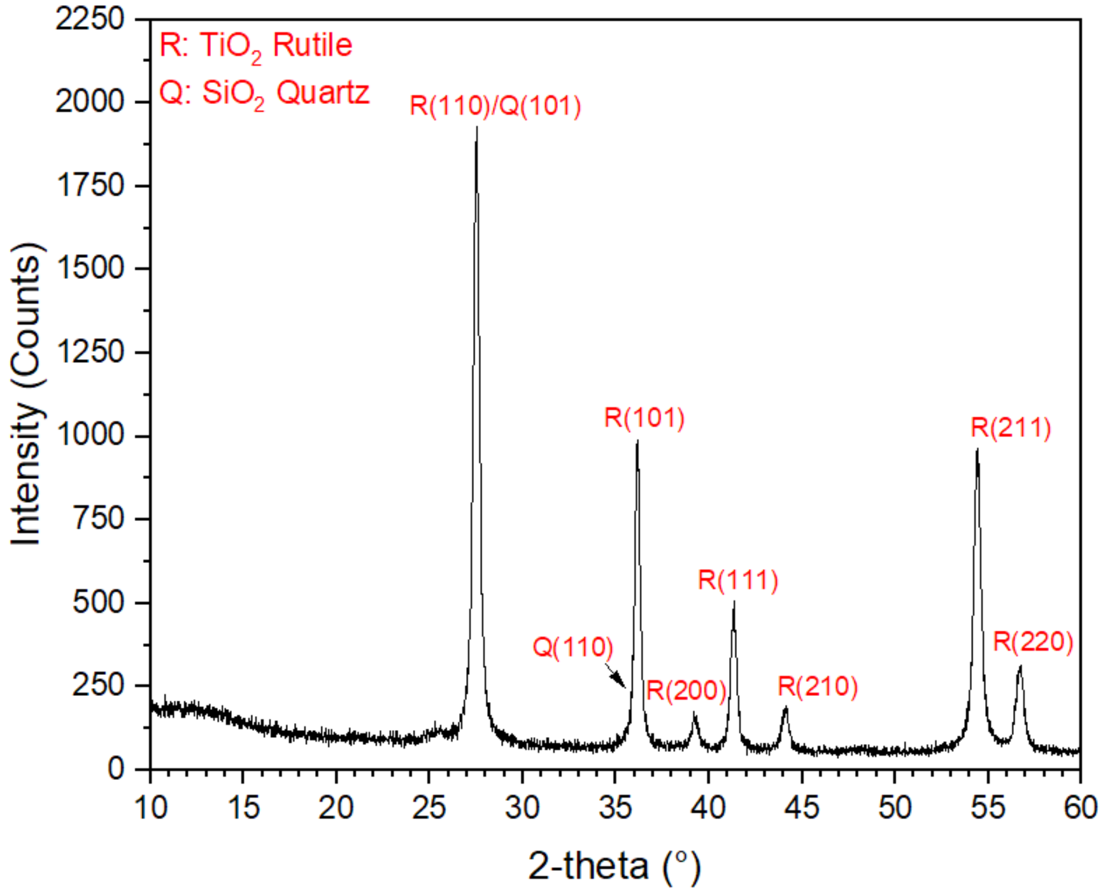

Figure S2. XRD spectrum of nTS.

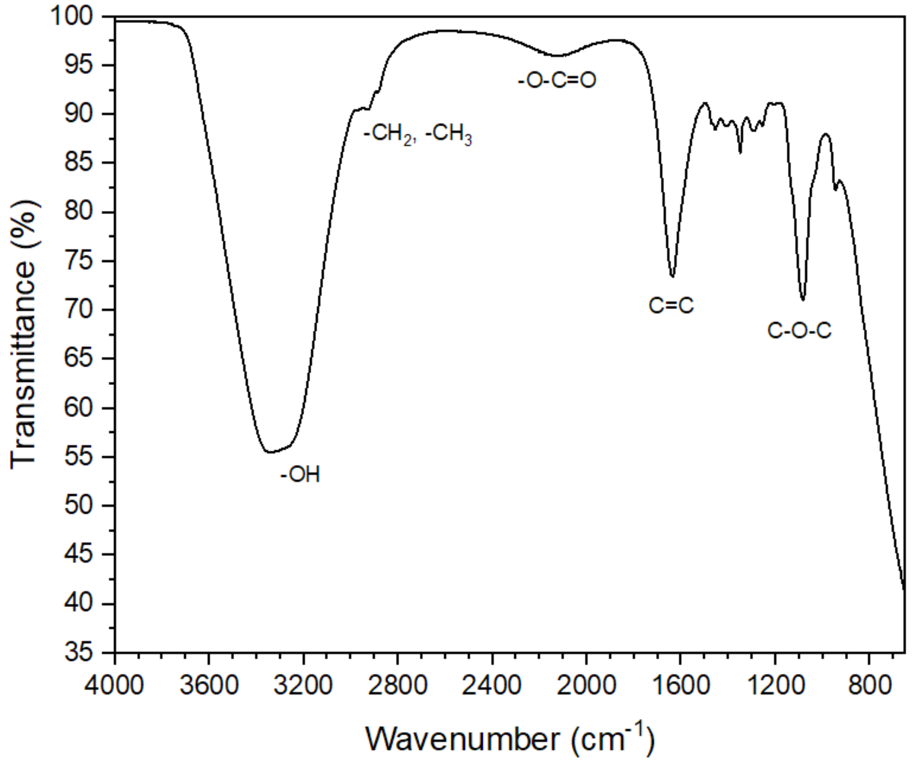

Figure S3. FTIR spectrum of PCE.

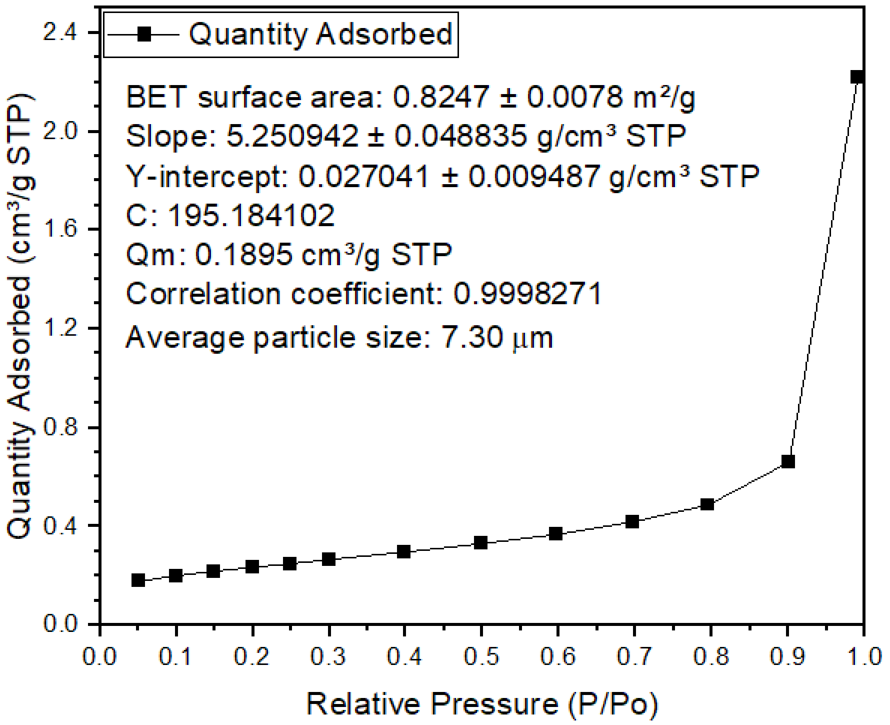

Figure S4. Isothermal graph of anhydrous cement class G.

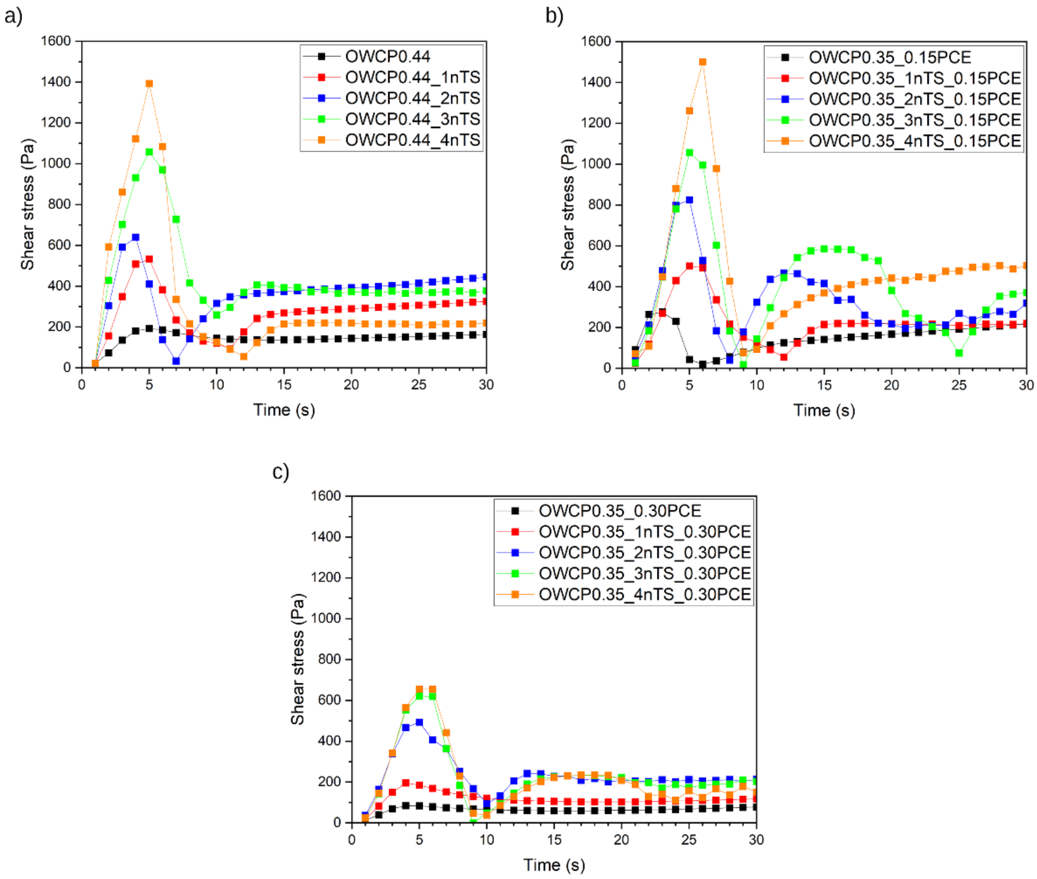

Figure S5. Shear stress graphs for statis yield stress determination at 25°C for the mixtures (**a**) without PCE, (**b**) with 0.15 wt.% of PCE, and (**c**) with 0.30 wt.% of PCE.

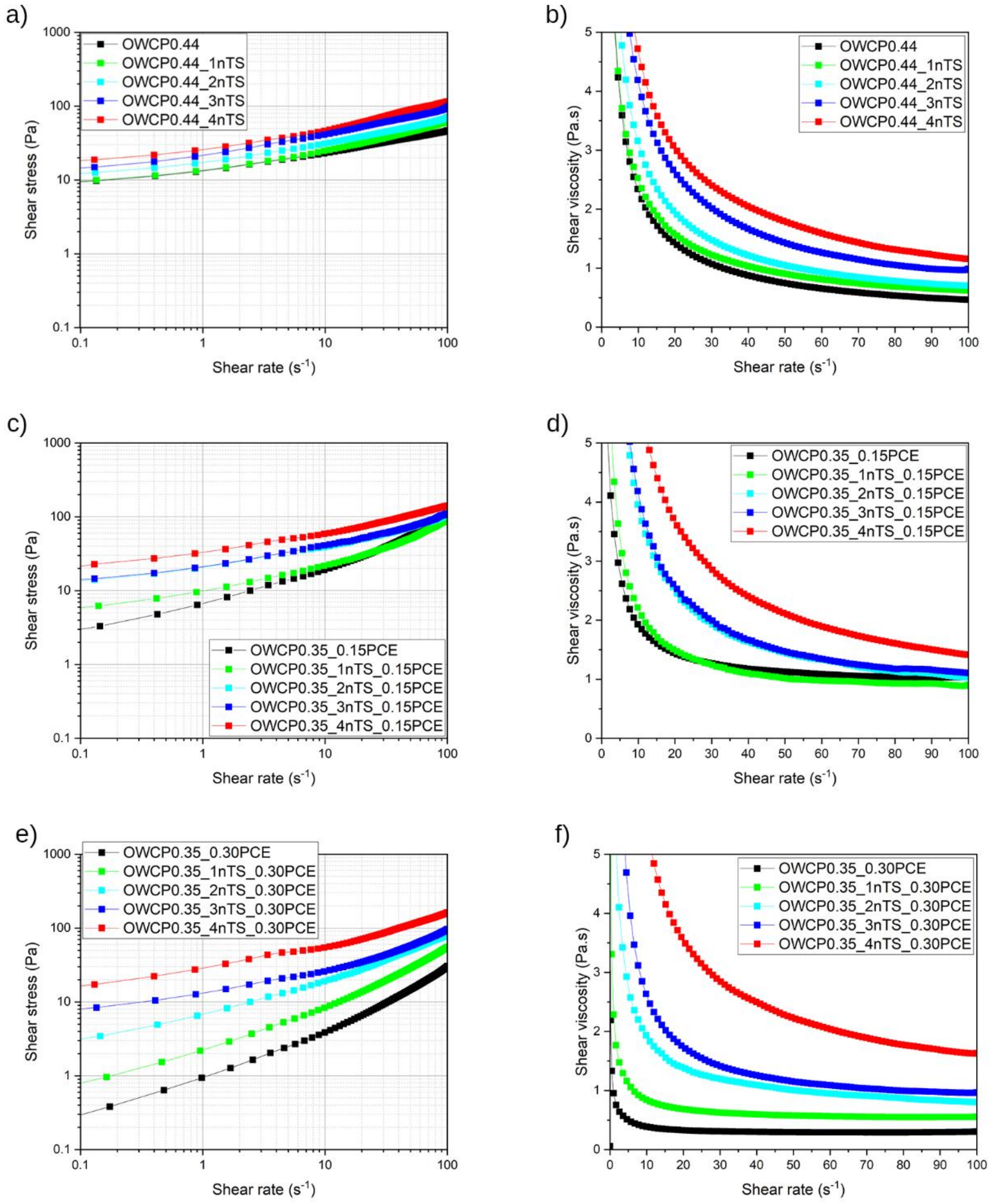

Figure S6. Shear stress and shear viscosity graphs at 25°C for the mixtures (**a**,**b**) without PCE, (**c**,**d**) with 0.15 wt.% of PCE, and (**e**,**f**) with 0.30 wt.% of PCE.

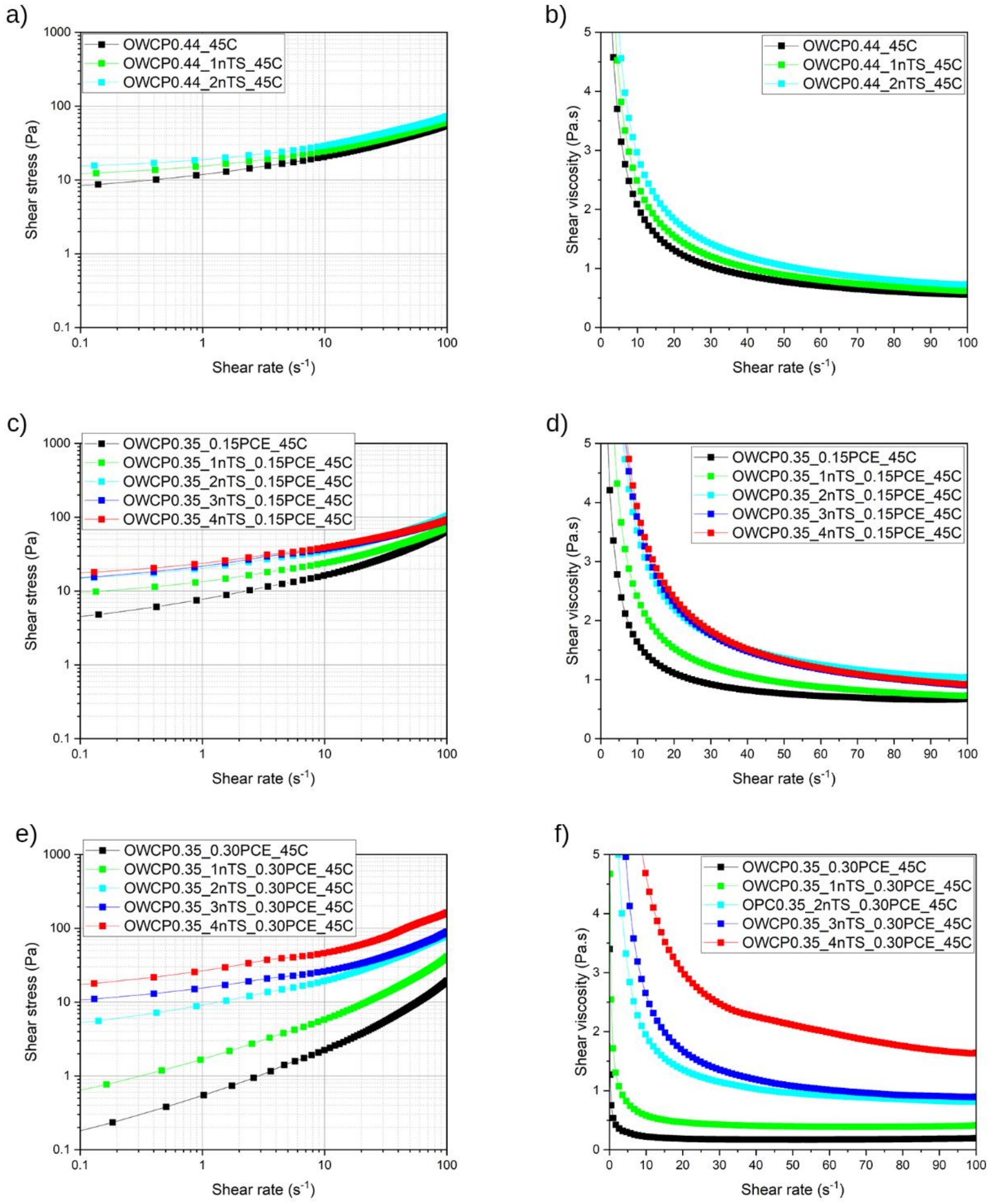

Figure S7. Shear stress and shear viscosity graphs at 45°C for the mixtures (**a**,**b**) without PCE, (**c**,**d**) with 0.15 wt.% of PCE, and (**e**,**f**) with 0.30 wt.% of PCE.

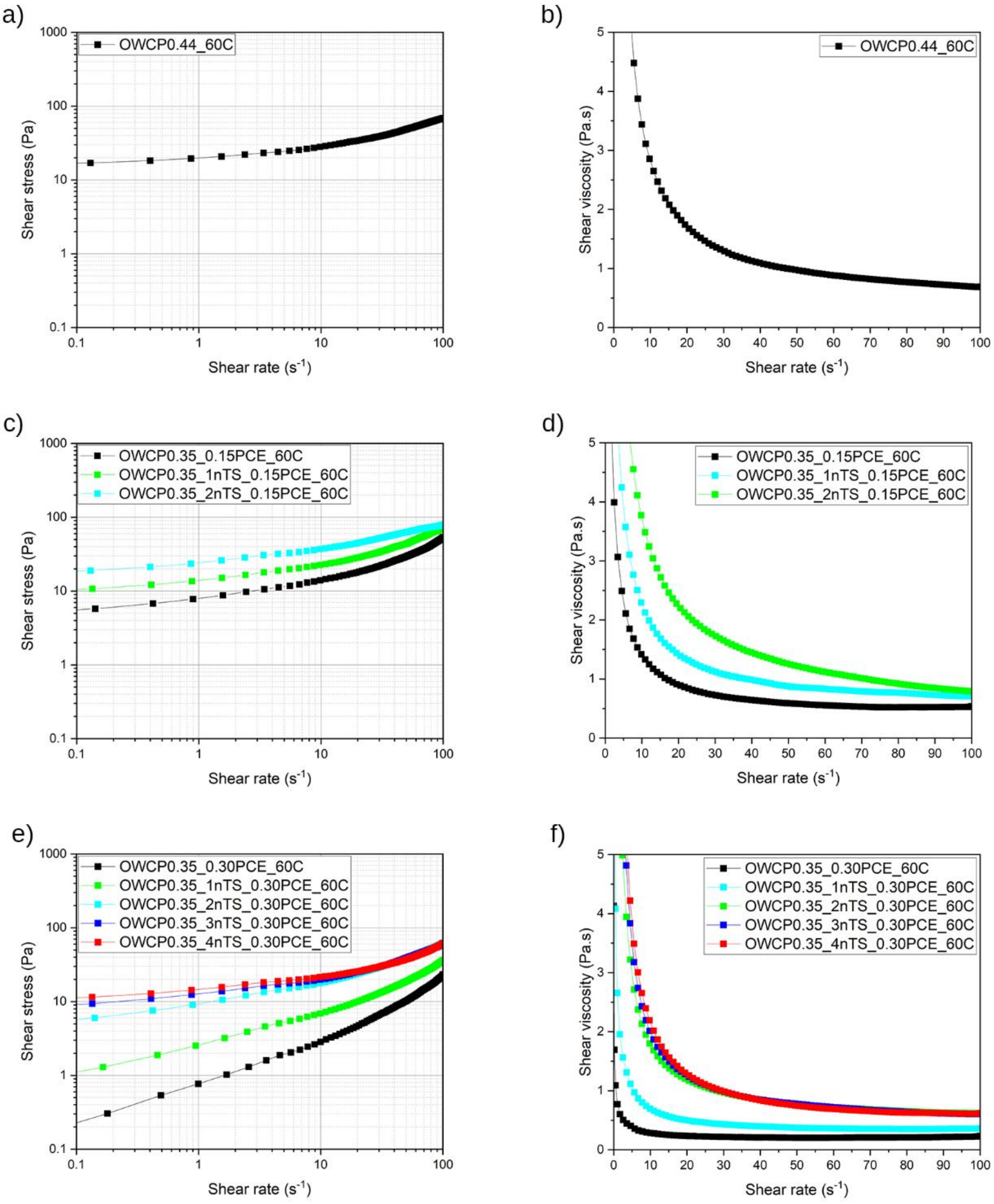

Figure S8. Shear stress and shear viscosity graphs at 60°C for the mixtures (**a**,**b**) without PCE, (**c**,**d**) with 0.15 wt.% of PCE, and (**e**,**f**) with 0.30 wt.% of PCE.
